# Supplementary material for: Effects of producer service industry co-agglomeration and manufacturing on the green and high-end transformation of manufacturing: An empirical study from Hunan Province
Source: PLoS One. 2024 Sep 6;19(9):e0297591. doi: 10.1371/journal.pone.0297591 (PMC11379371; doi:10.1371/journal.pone.0297591)
Supplement: Attachment — Submitted filename: Response letter.docx [file pone.0297591.s003.docx]

Reviewer #1: Following is the comment:

Some of the text in Table 1 is unclear, please adjust the format. And check the formatting of all tables in this article.

Reply: Dear, thanks a lot for your valuable suggestions. Yes, we have checked the formatting of all tables and adjusted the format of the tables.

Reviewer #2: The revisions of this paper have basically met the requirements. I agree to publish. Hope all the best

Reply: Dear, thanks a lot for your kind response !
